# Age-related accumulation of T cells with markers of relatively stronger autoreactivity leads to functional erosion of T cells

**DOI:** 10.1186/1471-2172-13-8

**Published:** 2012-02-09

**Authors:** Zohreh Tatari-Calderone, Milica Stojakovic, Ramita Dewan, Gama Le Bouder, Dragana Jankovic, Stanislav Vukmanovic

**Affiliations:** 1 Center for Cancer and Immunology Research, Children's Research Institute, Children's National Medical Center, Washington, DC, USA; 2 Sheikh Zayed Institute for Pediatric Surgical Innovation, Children's National Medical Center, Washington, DC, USA; 3 Immunobiology Section, Laboratory of Parasitic Diseases, National Institute of Allergy and Infectious Diseases, National Institutes of Health, Bethesda, MD, USA; 4 Sheikh Zayed Institute for Pediatric Surgical Innovation, Children's National Medical Center, 111 Michigan Avenue NW, Washington, DC 20010-2970, USA

## Abstract

**Background:**

Thymic involution is a prominent characteristic of an aging immune system. When thymic function is reduced/absent, the peripheral T cell pool is subject to the laws of peripheral T cell homeostasis that favor survival/expansion of T cell receptors with relatively higher functional avidity for self-peptide/MHC complexes. Due to difficulties in assessing the TCR avidity in polyclonal population of T cells, it is currently not known whether high avidity T cells preferentially survive in aging individuals, and what impact this might have on the function of the immune system and development of autoimmune diseases.

**Results:**

The phenotype of T cells from aged mice (18-24 months) indicating functional TCR avidity (CD3 and CD5 expression) correlates with the level of preserved thymic function. In mice with moderate thymic output (> 30% of peripheral CD62L^hi ^T cells), T cells displayed CD3^low^CD5^hi ^phenotype characteristic for high functional avidity. In old mice with drastically low numbers of CD62L^hi ^T cells reduced CD5 levels were found. After adult thymectomy, T cells of young mice developed CD3^low^CD5^hi ^phenotype, followed by a CD3^low^CD5^low ^phenotype. Spleens of old mice with the CD3^low^/CD5^hi ^T cell phenotype displayed increased levels of IL-10 mRNA, and their T cells could be induced to secrete IL-10 in vitro. In contrast, downmodulation of CD5 was accompanied with reduced *IL-10 *expression and impaired anti-CD3 induced proliferation. Irrespective of the CD3/CD5 phenotype, reduced severity of experimental allergic myelitis occurred in old mice. In MTB TCRβ transgenic mice that display globally elevated TCR avidity for self peptide/MHC, identical change patterns occurred, only at an accelerated pace.

**Conclusions:**

These findings suggest that age-associated dysfunctions of the immune system could in part be due to functional erosion of T cells devised to protect the hosts from the prolonged exposure to T cells with high-avidity for self.

## Background

Immune system of elderly displays complex set of changes relative to young individuals. Of the many variations observed, altered T cell function is the most consistent and most dramatic one [1]. Despite relatively normal numbers of CD4^+ ^and CD8^+ ^lymphocytes, T-cell dependent functions of the immune system of aged individuals are defective, as evidenced by reduced DTH reactions and antibody production in response to vaccination and infection [2]. This could be due to reduced proliferation of T cells, evident at biochemical level by defects in proximal TCR signaling cascade activation [3-5] and calcium signaling [6], and at cellular level by defects in cytokine production [7] and differentiation to Th1 or Th2 effector cells [8]. In addition, the frequency of antigen-specific naive T cells is reduced, due to an impaired production of T cells caused by thymic involution [9]. Reduction of the thymus size and cellularity generally starts in puberty [10] and is thought to reflect depletion of thymic stromal tissue [11], as well as thymus repopulation by bone marrow derived precursors [12].

The lower supply of naive T cells leads to a shift in the balance between memory and naive T cells, with overrepresentation of the former [3]. This is due to transitioning of the naive into the memory T cells following activation with antigen, but also in response to the laws of T cell homeostasis. The peripheral T cell pool is maintained by production of new T cells by the thymus, and homeostasis-driven expansion of peripheral T cells [13]. If the function of thymus is reduced, the numbers of T cells are maintained by a compensatory increase in homeostatic expansion [14,15]. The extent of homeostatic expansion of any given T cell is dependent on the functional avidity of the TCR for self-peptide/MHC complexes [16-18]. One would therefore predict that the peripheral repertoire of T cells would skew towards high avidity T cells sometime following age-associated thymic involution. This issue, however, has not been addressed to date, due to difficulties in assessing the TCR avidity in polyclonal population of T cells. T cells constantly tune their sensitivity to self-peptide/MHC complexes by changing the levels of TCR/CD3 and CD5 molecules [19-21]. The letter is an inhibitor of TCR signaling [22], and T cells perceiving strong signals up-modulate CD5 to reduce signaling and avoid over-stimulation. Similar impact is achieved by down-modulating CD3, producing a CD3^lo^CD5^hi ^phenotype. Converse phenotypic changes occur if surrounding signals are perceived weak- T cells increase their sensitivity by up-regulating CD3 levels and down-modulating CD5, producing a CD3^hi^CD5^lo ^phenotype. Thus, CD3 and CD5 levels can serve as indicator of the strength of signal perception by T cells, and if the levels of self-peptide/MHC are constant, the major determinant of the signal magnitude generated is the affinity/avidity of the TCR for self-peptide/MHC complexes. Taking advantage of the fact that levels of CD5 and TCR/CD3 expression can be used to predict relative TCR avidity [21], we have shown that relative levels of these two molecules could be used as markers of overall avidity of the TCR repertoire [23].

The avidity of TCR for antigen is considered an important characteristic of an efficient T cell-mediated immunity [24]. Protection against tumors or infectious agents by monoclonal (or oligoclonal) T cell populations correlates with the avidity of the TCR for given antigen [25,26]. However, increase in the avidity to foreign antigens also elevates the avidity to self-peptide/MHC complexes [27], likely due to cross-reactivity between cognate antigen and self-peptides that promote selection and homeostasis of T cells [28]. As with foreign antigens, higher avidity for auto-antigens leads to more severe autoimmunity [29-32]. Furthermore, high avidity TCR engagement with self peptide/MHC molecules may induce both cell intrinsic and extrinsic compensatory mechanisms [33], rendering it impossible to predict the net effect of high avidity TCR recognition on the function of a polyclonal immune system. We here show that CD3/CD5 phenotype of T cells undergoes a two-phase change in aged, as well as mice subjected to adult thymectomy. Increased CD5 and reduced CD3 levels in the first phase are consistent with preferential survival of high-avidity T cells, while the second phase was characterized by a drop in CD5 levels. The latter change appears to be a part of a shut-down of T cell function, that followed a period with increased production of IL-10. We speculate that this may be an attempt to counteract the preferential survival of high avidity T cells due to their potential to induce stronger autoimmune responses.

## Results

### Dynamics of peripheral T cell CD3 and CD5 levels in the function of thymic export

**Figure 1:**
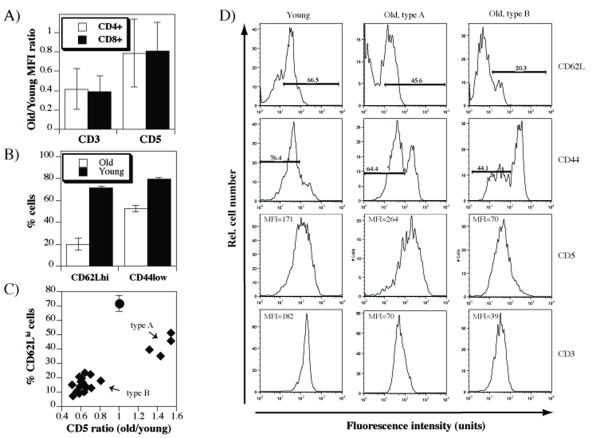
**Changes of CD3 and CD5 levels in old mice**. **A**) Spleen cells from young (8-12 weeks old) and old (12-24 months old) mice were stained with anti-CD4-PE, anti-CD3-FITC and anti-CD5-APC, or anti-CD8-PE, anti-CD3-FITC and anti-CD5-APC antibodies and analyzed by flow cytometry. Shown are ratios of CD3 or CD5 mean fluorescence intensities obtained on gated CD4^+ ^or CD8^+ ^spleen cells from matched old and young individual mice. **B**) Spleen cells from the same set of mice were stained with anti-CD4-PE, anti-CD62L-FITC and anti-CD44-APC, or anti-CD8-PE, anti-CD62L-FITC and anti-CD44-APC antibodies and analyzed by flow cytometry. Shown are % of CD62L^hi ^or CD44^hi ^cells on gated CD4^+ ^spleen cells. Similar results were seen in CD8^+ ^T cells (data not shown). **C**) Dot plot showing relationship between %CD62L^hi ^cells found in spleens of old mice and the ratio of CD5 mean fluorescence intensities (CD4^+ ^T cells in old/CD4^+ ^T cells in young mice). As a reference, the value found in young mice is displayed as closed circle. Two cohorts, each consisting of 10 old mice were compared to two sets of 10 young mice and cumulative data is shown. D) Shown are representative histograms of CD3, CD5, CD44, and CD62L staining on gated CD4^+ ^T cells analyzed in A-C, depicting the two patterns of CD5 expression that appear in old mice.

To determine whether the level of thymic output in old mice affects the functional avidity of the peripheral TCR repertoire, we compared the levels of CD3 and CD5 cell surface molecules on T cells of young (8-12 weeks old) and old (18-24 months old) mice. Because of day-to-day experimental variations and the use of antibodies conjugated to different fluorochromes, we calculated ratios of mean fluorescence intensities (MFI) obtained in spleen cells from paired old and young mice, as previously described [23]. Significant (*p *< 0.0001) reduction of CD3 levels was observed in all old mice, while the levels of CD5, although overall significantly (*p *= 0.0127) reduced, were heterogenous (Figure 1A). These differences were observed in both CD4^+ ^and CD8^+ ^T cells. Expression of CD62L and CD44 were taken as indicators of preserved thymic output in individual old mice (Figure 1B). These parameters revealed a correlation between relatively well-preserved thymic export (> 30% CD62Lhi cells) and the upregulation of CD5 on peripheral T cells (Figure 1C). The individual old mice carrying T cells with higher levels of CD5 are herewith referred to as "type A", while the mice with T cells having lower levels of CD5 than the control mice as "type B".

**Figure 2:**
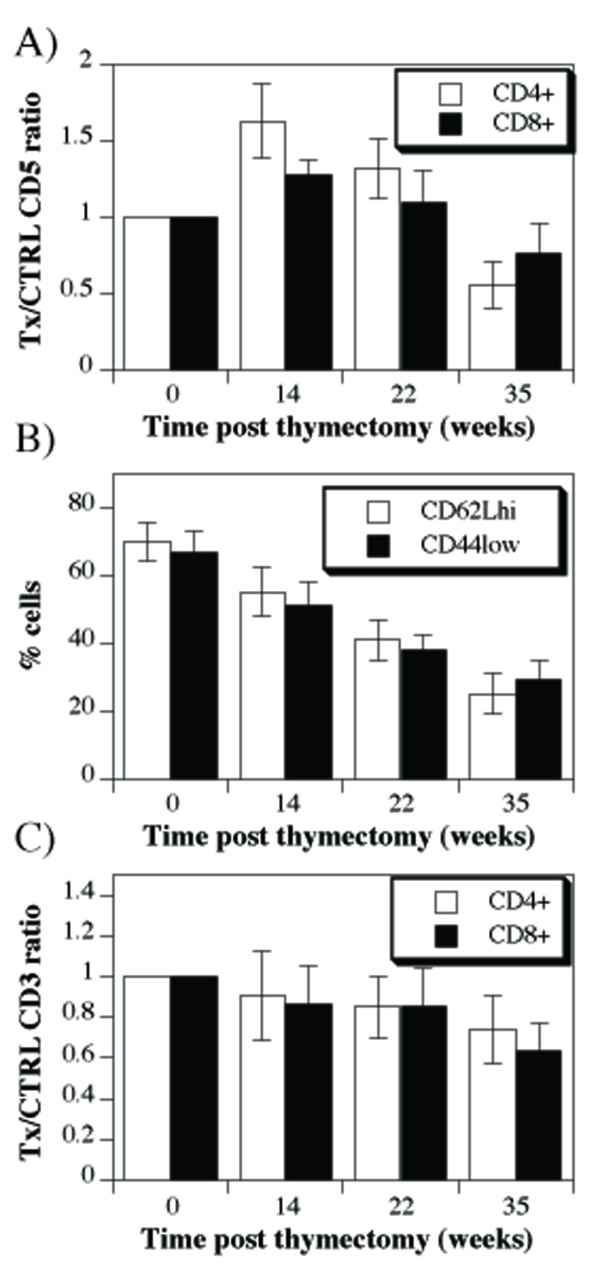
**Changes of CD3 and CD5 levels following adult thymectomy**. **A**) Spleen cells from control and adult thymectomized mice were stained with anti-CD4-PE, anti-CD3-FITC and anti-CD5-APC, or anti-CD8-PE, anti-CD3-FITC and anti-CD5-APC antibodies and analyzed by flow cytometry. Shown are ratios of CD5 mean fluorescence intensities obtained on gated CD4^+ ^or CD8^+ ^spleen cells from matched control and thymectomized individual mice (four mice per group each time point). **B**) Spleen cells from the same set of mice were stained with anti-CD4-PE, anti-CD62L-FITC and anti-CD44-APC antibodies and analyzed by flow cytometry. Shown are percent CD62L^hi ^or CD44^hi ^cells on gated CD4^+ ^spleen cells. **C**) Shown are ratios of CD3 mean fluorescence intensities obtained on gated CD4^+ ^or CD8^+ ^spleen cells from matched control and thymectomized individual mice, stained as described in (A)

Since the thymic function progressively declines with age, development of "type A" likely precedes that of "type B" phenotype. This, however, is difficult to verify due to individual variations in the rate of thymic involution. To determine the sequence of phenotypes directly, we performed adult thymectomy and followed the phenotype of T cells. These results clearly indicated that the changes in the levels of CD5 expression occur in two phases: phase with increased levels of CD5 precedes the one with low CD5 levels (Figure 2A). The phenotypic switch occurred following a drastic reduction of T cells with naive phenotype similar to the one seen in old mice (Figure 2B). In contrast to the CD5, the levels of CD3 showed mild, but a progressive decline (Figure 2C).

### Distinct functional phenotypes of T cells from type A- or type B- old mice

**Figure 3:**
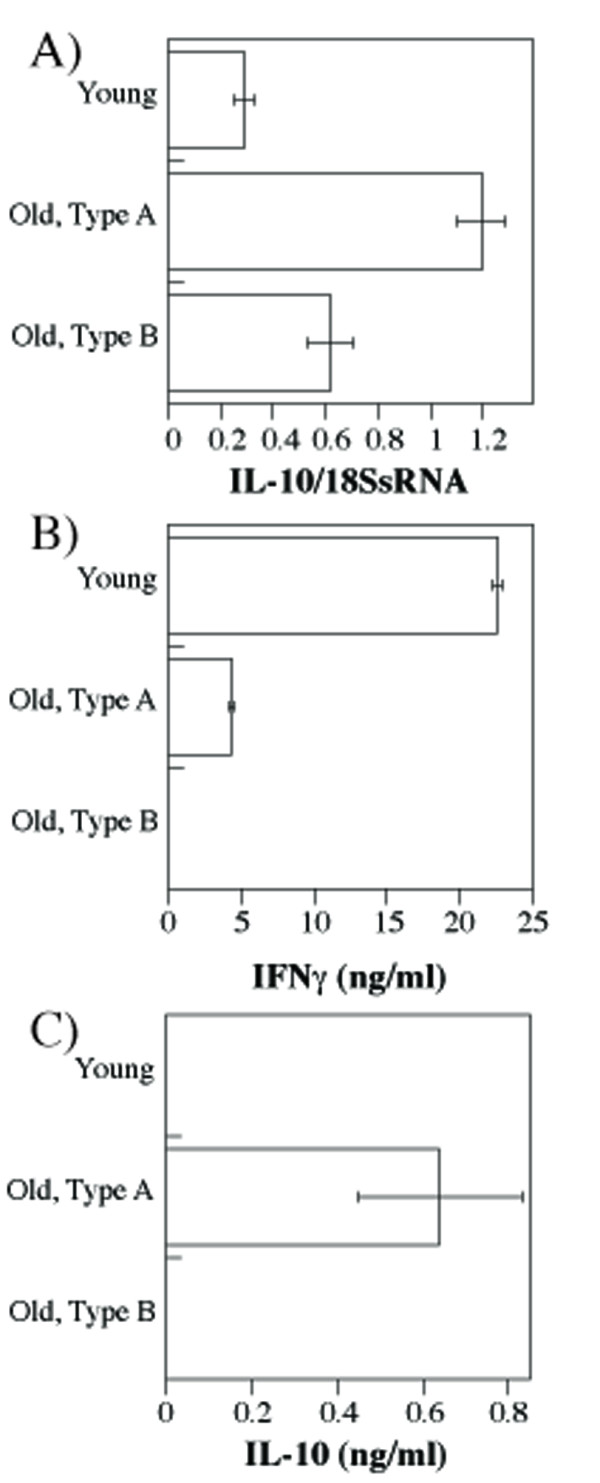
**Age-dependent increase in IL-10 production**. **A**) Total cellular RNA isolated from the spleens of young and old mice was used to obtain cDNAs which served as templates for the real time PCR using IL-10-specific primers. Shown are means and standard errors of IL-10 mRNA quantities relative to the 18S rRNA for triplicate reactions of each sample for three individual mice per group. **B**-**C**) Spleen cells from young or old mice (N = 3) were stimulated with plate-bound anti-CD3 antibody. Following 3 day culture, cells were stimulated with PMA/ionomycin and supernatants were tested for interferon-γ (B) or IL-10 (C) content by ELISA. No IL-10 protein was detected in unstimulated control cells (data not shown)

Lower levels of CD3 and higher levels of CD5 are indicators of high functional TCR avidity [21,23], and chronic high-avidity TCR stimulation leads to production of IL-10 [34-37]. We therefore sought to determine whether aging immune system is prone to produce IL-10. IL-10 mRNA was significantly increased in the spleens of old relative to young mice (Figure 3A). Stimulation of T cells in vitro showed the propensity of young mice to respond by IFNγ production (Figure 3B), while T cells from type A mice produced IL-10 (Figure 3C), as well as limited amounts of IFNγ. Interestingly, T cells from type B old mice produced neither IFNγ nor IL-10.

### Reduced T cell responses of old mice in vitro and in vivo

**Figure 4:**
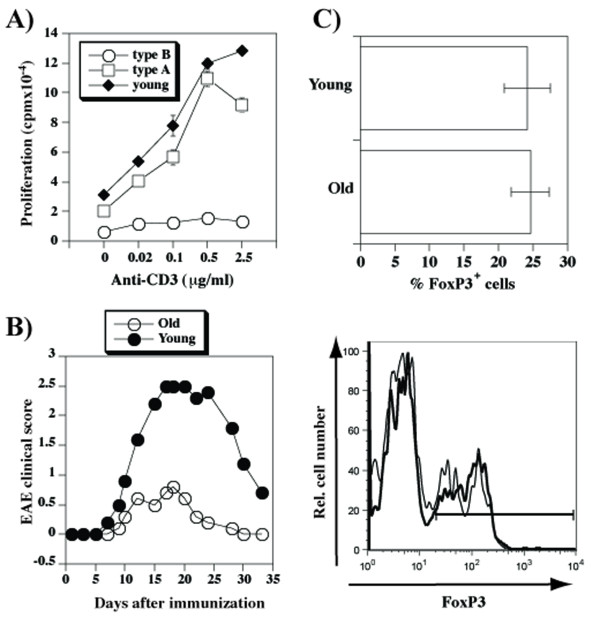
**Reduced T cell responsiveness in aged mice**. **A**) Anti-CD3-induced proliferation (means ± SD) of spleen cells from young and old WT mice. Shown are values from individual mice from a representative of four separate experiments. **B**) EAE was induced in 3 (closed symbols) or 16 (open symbols) months old female WT mice. Shown are mean clinical scores of groups of ten mice each. **C**) Spleen cells from young (8-12 weeks old) and old (12-24 months old) mice were stained with anti-CD4 (extracellular stain) and anti-FoxP3 (intracellular stain). Shown are mean (± SD) percentage of FoxP3^+ ^cells within gated CD4^+ ^populations (upper panel), and representative histograms for young (plain line) or old (bold line) mice.

Inability of type B old mice to produce IFNγ suggested that T cells from these mice may be hyporesponsive. To test this possibility we evaluated proliferation of T cells to anti-CD3 stimulation. As expected, the response was dramatically reduced in type B, but not type A old mice (Figure 4A). To evaluate whether aging affects T cell responses to antigen in vivo, we induced experimental allergic encephalomyelitis (EAE) and found that clinical signs of the disease were significantly less severe in old than in the young mice (Figure 4B), irrespective of whether their T cells displayed type A or type B CD3/CD5 phenotype after the end of the observation period (data not shown). The reduced responses in old mice occurred in the presence of similar numbers of natural regulatory T cells (Figure 4C). Thus, at least two distinct mechanisms (propensity to secrete IL-10 in "type A" and globally reduced responsiveness in "type B") contribute to reduced susceptibility of old mice to EAE.

### Age related changes in T cells are accelerated in mice with increased avidity for self

**Figure 5:**
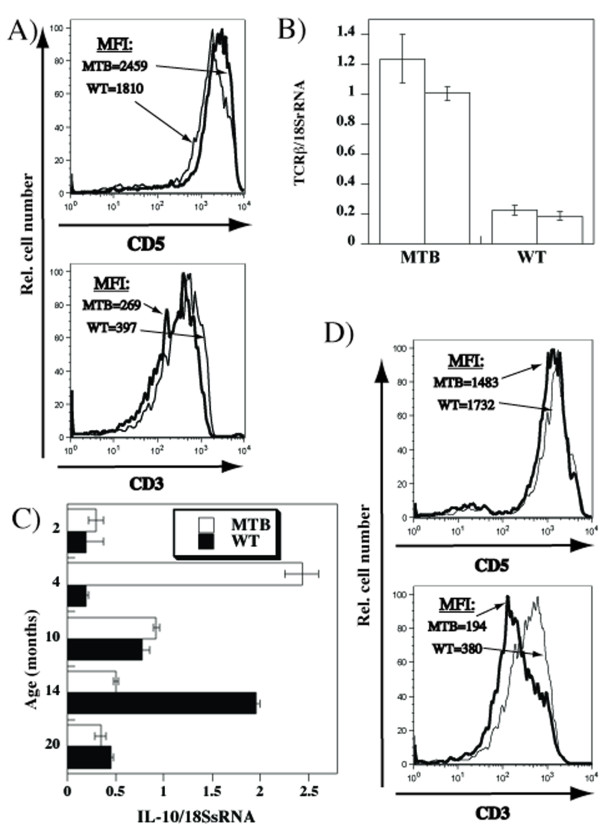
**Earlier onset of age dependent changes in CD3/CD5 phenotype and IL-10 induction in MTB mice**. **A**) Spleen cells from 7 week old WT or MTB mice were stained with anti-CD4-FITC, anti-CD5-APC and anti-CD3-PerCP antibodies and analyzed by flow cytometry. Shown are overlay histograms of CD5 or CD3 expression in WT (plain line) and MTB (bold line) CD4^+ ^cells. The numbers indicate mean fluorescence intensities. **B**) cDNAs obtained from 12 weeks old MTB or WT spleens served as templates for real time PCR using TCRβ-specific primers. Shown are means and standard errors of TCRβ mRNA relative to the 18S rRNA for triplicate reactions of each sample for two individual mice of each genotype. Total cellular RNA isolated from the spleens of MTB or WT mice was used to obtain cDNAs which served as templates for the real time PCR using IL-10-specific primers. Shown are means and standard errors of IL-10 mRNA quantities relative to the 18S rRNA for triplicate reactions of each sample. **D**) CD5 or CD3 expression in 10 months old WT (plain line) and MTB (bold line) CD4^+ ^spleen cells labeled with anti-CD4-FITC, anti-CD5-APC and anti-CD3-PerCP antibodies. The numbers indicate mean fluorescence intensities.

If the age-associated increased production of IL-10 and subsequent crush in T cell function are a consequence of preferential survival and/or homeostatic expansion of T cells with high avidity for self, then the observed changes should occur prematurely if the avidity of T cells is artificially raised. MTB TCRβ transgenic mice display globally increased avidity for peptide/MHC complexes due to stronger interactions with MHC molecules [23]. As previously reported, higher levels of CD5 with fewer cell surface TCR/CD3 were found in MTB than in WT mice (Figure 5A). The TCR/CD3 levels were reduced despite about five fold higher levels of mRNA encoding the constant region of TCRβ (Figure 5B), arguing against the explanations related to the transgene expression. As predicted, we found that increases in the IL-10 mRNA peaked much earlier in MTB than in the WT mice (Figure 5C). The subsequent decrease occurred also earlier in MTB mice. This decrease in IL-10mRNA levels was associated with a more pronounced reduction of cell surface TCR/CD3 levels, and a reverse trend in CD5 expression (Figure 5D).

**Figure 6:**
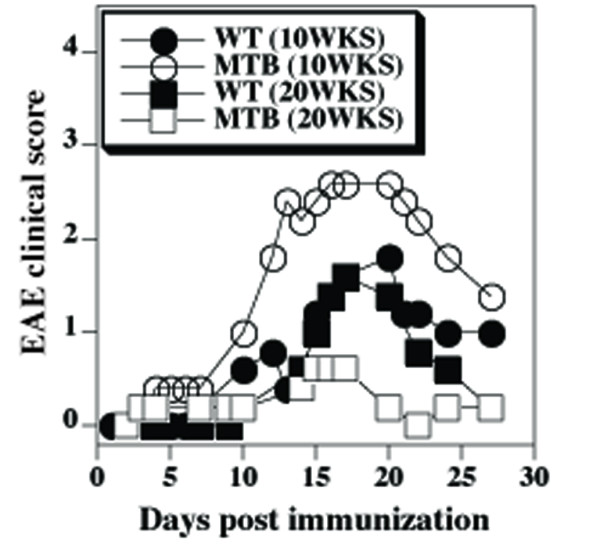
**Earlier onset of age-associated reduced severity of EAE in MTB mice**. Mean clinical scores of EAE in MTB and WT mice (five each) immunized at 10 or 20 weeks of age with MOG_38-50 _peptide.

**Figure 7:**
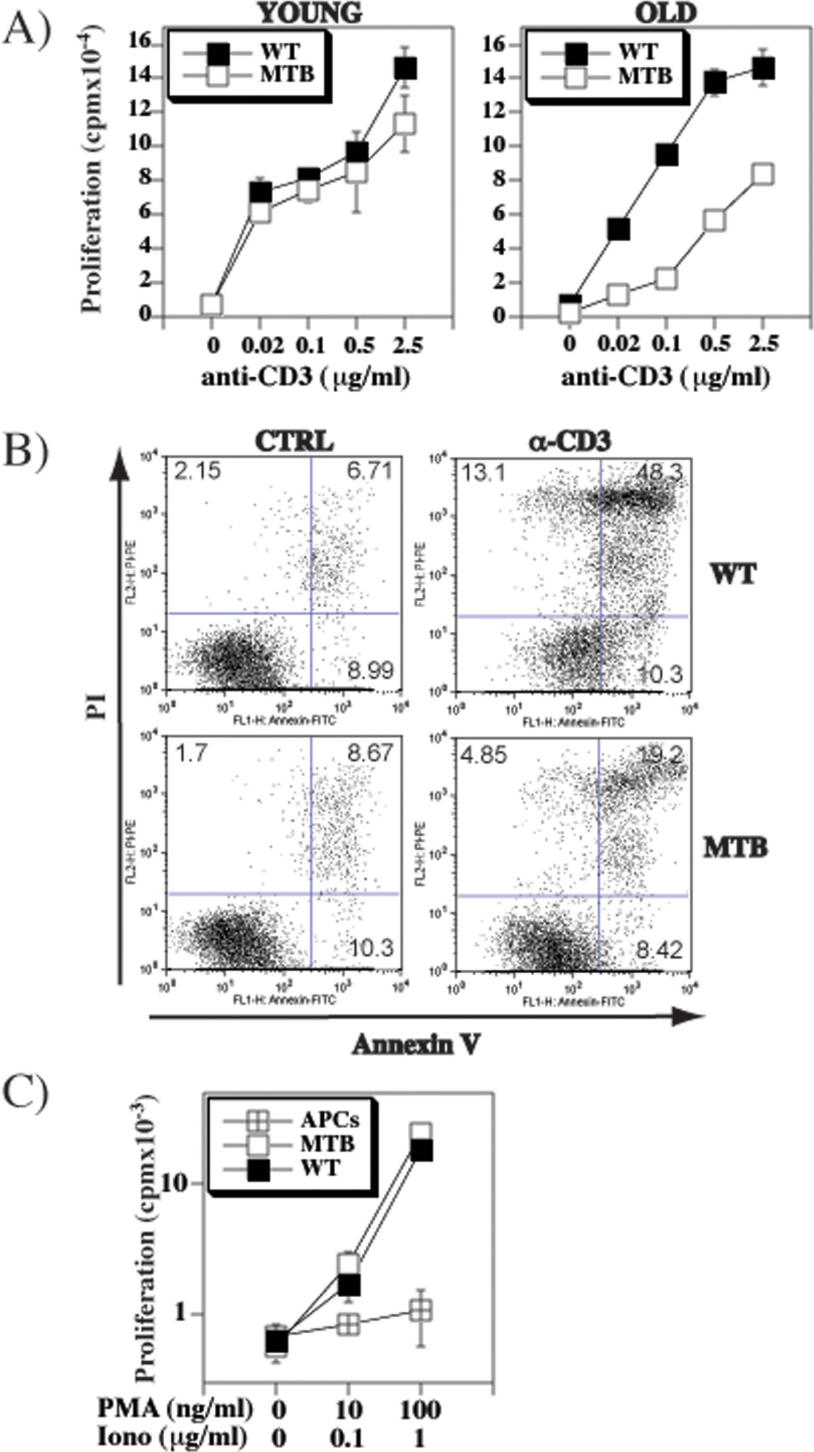
**Earlier onset of in vitro T-cell disfunction in MTB mice**. **A**) Anti-CD3-induced proliferation (means ± SD) of spleen cells from 7 weeks (young) or 17 months old (old) WT or MTB mice. **B**) Spleen cells from 20 weeks old MTB and WT mice were cultured in the presence or absence of anti-CD3 antibody for 3 days. Following subsequent 24 hour stimulation with anti-CD3, cells were stained with anti-annexin V and propidium iodide. **C**) Proliferation to PMA/ionomycin of purified T cells from 15 months old MTB or WT mice in the presence of irradiated WT antigen presenting cells.

As expected, changes in T cell phenotype and IL-10 mRNA levels in MTB mice coincided with reduced severity of EAE in 20 weeks old MTB mice relative to their WT couterparts (Figure 6). In contrast, young MTB mice (10 weeks old) developed a more severe form and an earlier onset of the disease than the WT mice. The stronger disease in young MTB mice occurred despite relatively mild defect of in vitro T cell responses to anti-CD3 stimulation (Figure 7A). This finding is consistent with relatively higher avidity of MTB T cells for self-peptide/MHC complexes. As the MTB mice aged, the defect in anti-CD3 induced T cell responses became more profound (Figure 7A), consistent with down-modulation of cell surface CD3.

Defective responses of T cells with down-modulated CD5 could be a result of increased propensity for activation induced cell death. We therefore compared the levels of apoptosis following anti-CD3 stimulation. Reduced levels of apoptosis were observed in MTB relative to the WT T cells (Figure 7B), suggesting that activation induced cell death is not a likely cause of reduced T cell responses in T cells with decreased CD5 levels.

To determine whether the hyporesponsiveness is due to general inability of T cells to respond or due to TCR signaling defect, we subjected T cells to pharmacologic stimulation consisting of phorbol esters and ionomycin, that by-passes the most proximal TCR signaling. The results show relatively similar potential of cells to respond to this stimulation (Figure 7C), suggesting that the cause of hyporesponsiveness is related to the CD3/TCR complex-mediated signal transduction.

## Discussion

Our findings demonstrate that following a shortage in supply of newly developed T cells due to thymic involution, peripheral T cell compartment in aged mice displays a sequence of phenotypic and functional changes important for the functioning of the entire immune system. These changes follow a characteristic pattern. The first phase was predictable by the known effects of T cell homeostasis in lymphopenic conditions and the effect of the functional avidity of the TCR on peripheral T cell homeostasis. In this phase, there was an increase of both CD4^+ ^and CD8^+ ^T cells with a phenotype (CD3^low^CD5^hi^) indicative of relatively higher functional avidity for self-peptide/MHC complexes [21]. The second phase is characterized by development of functional erosion of T cells, caused by a defect in TCR-mediated activation.

Self-peptide/MHC complexes are involved in many aspects of T cell physiology, promoting thymocyte differentiation [38,39], enabling peripheral survival and homeostasis of naïve T cells and modulating their activation by antigens [40-42]. Under lymphopenic conditions residual T cells proliferate to reconstitute their nearly normal numbers [41]. T cells with higher avidity for self-peptide/MHC complexes enjoy advantage and expand more relative to the low avidity T cells [16-18]. During and or consequent to the expansion process T cells acquire phenotype of activated/memory T cells and acquire effector functions [43-47]. In addition to the faster disappearance of T cells with naive phenotype, the accumulation of high avidity T cells and their partial activation is potentially dangerous due to increased risk of autoimmune disorders. In fact, lymphopenic conditions are known to be associated with autoimmune phenomena [48]. However, the incidence of autoimmune diseases in general does not increase in elderly, despite development of lymphopenia.

In humans, each autoimmune disease has a characteristic pattern of incidence. Although average peak of incidence differs for each individual autoimmune disease, a general trend suggests that most autoimmune diseases develop either during puberty (juvenile type diseases) or during mature reproductive life of individuals. For example, lupus erythematosus affects primarily women of childbearing age, and most frequently begins between ages of 15 and 40 years [49]. The average age of onset of multiple sclerosis is 28-30 years [50]. The number of new cases in both diseases, as well as other autoimmune diseases, reduces with further age. What could be the reason for this decline? The function of the immune system declines with aging in both mice and humans, limiting its ability to respond to infections and vaccines [2,15]. The changes are mainly due to dysfunctions in the T cell compartment while the activity of B cells and innate immunity are less affected [2,51]. However, these changes occur at age of 70 or higher, and are unlikely responsible for the decline in incidence of autoimmunity after the ages of 30-40. Our results showing attenuated clinical EAE in aged mice are in agreement with the incidence of human autoimmune disorders. Concomitant changes in T cell phenotype and function of old mice suggest that reasons for reduced autoimmunity may be intrinsic to T cells. This notion is further supported by an earlier occurrence of EAE attenuation in MTB TCRβ transgenic mice, since the transgene is expressed by T cells and hence affects primarily the function of T cells. Interestingly, we have previously shown an age-dependent arrest in the progression of lupus in F1 offsprings of MTB and lupus-prone BXSB strain [52]. This arrest was coupled with reduced activation of T cells in vivo and reduced numbers of natural regulatory T cells, suggesting again a mechanism intrinsic to T cells. The numbers and function of natural regulatory T cells in MTB mice on B6 background (used in the present study) are indistinguishable from the WT mice [23], arguing against the role of these cells in reduced susceptibility of MTB mice to EAE. It remains to be determined whether intrinsic mechanisms additional to the two identified here (increased IL-10 production and functional arrest) may be involved, such as possibly changes in IL-17 production- a cytokine important for development of EAE [53].

It is tempting to speculate that the strategy of the immune system to counteract age associated increased risk of autoimmunity is promotion of differentiation of T cells with a potential to secrete IL-10 (so called Tr1 cells). In support of this notion, development of multiple sclerosis in humans is associated with defective development of Tr1 cells that secrete IL-10 [54,55]. IL-10 secretion as a result of chronic high-avidity TCR engagement has been described in other experimental models [34-37], and increased IL-10 production associated with aging has been reported in both aged mice [56] and humans [51]. Our results showing an earlier onset of IL-10 mRNA levels in mice with artificially higher TCR avidity for self-peptide/MHC complexes clearly supports this possibility, although this may not be the only mechanism affecting the function of the immune system in type A mice. Subsequent reduction, however, suggests that the control of enhanced T cell reactivity for self by IL-10 is temporary, and persisting chronic stimulation leads to a functional shut down.

Because IL-10-deficient mice develop enterocolitis [57], IL-10 is thought to be involved in maintenance of tolerance to self. However, IL-10 can also exert immunostimulatory properties, such as stimulation of B cell proliferation and differentiation into the antibody-secreting cells, and differentiation of CD8^+ ^T cells into effector cells [58]. Despite these stimulatory functions of IL-10, the effect of IL-10 in most studies of autoimmune diseases is one of regulation. Thus in EAE, systemic administration of IL-10 prior to EAE induction prevents the development of the disease [59,60]. In contrast to the actively induced EAE, injection of IL-10 exacerbated adoptively transferred form of the disease [61]. Removal of IL-10 by gene inactivation increases the severity of the disease [62-64], suggesting that IL-10-production has a physiological role in dampening the course of the EAE. In lupus, the levels of IL-10 found in the serum of affected patients correlate with the disease activity [65]. This could suggest involvement of IL-10 in the pathogenesis of the disease, but also (apparently unsuccessful) attempts of the immune system to regulate the ongoing autoimmune response. The former possibility is supported by ameliorating effects of anti-IL-10 antibody treatment in lupus patients [66], as well as in NZB hybrid mice [67]. However, these early results were countered with the findings of new studies. Thus, genetic deficiency of IL-10 resulted in significantly enhanced disease, while the treatment with recombinant IL-10 ameliorated the disease in the MRL model [68]. Furthermore, continuous low levels of IL-10 achieved by gene therapy approach also diminished the disease activity in NZB hybrid congenic mouse model [69]. Therefore, the exact role of IL-10 in lupus remains to be established.

T cell dysfunction resulting in progressive difficulties to raise immune responses have been described in elderly humans and mice [15,70]. Therefore, these findings suggest that T cell dysfunctions associated with aging can at least partly be explained by adaptive alterations in high-avidity T cells caused by their autoreactivity. Our findings parallel those of tumor infiltrating T cells that become non-functional if their TCR is of high, but not low affinity for antigen [71]. Thus, while their immediate impact may be effective, high avidity T cells may not be most desirable for long-term protection and/or preservation of immunological memory, as they are likely to functionally erode earlier than the low avidity T cells.

## Conclusions

**Figure 8:**
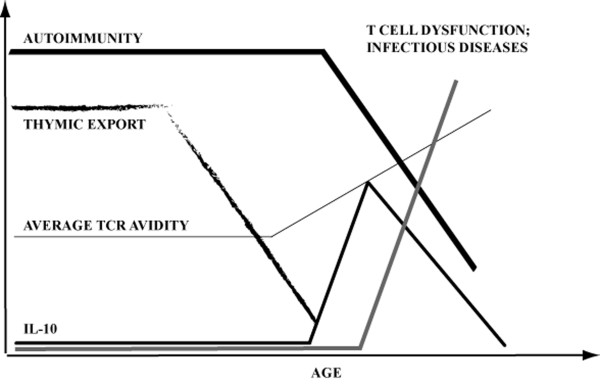
**Summary and implications of the findings in the present manuscript**.

T cell function deteriorates with age, leading to increased susceptibility of elderly to infections and higher incidence of cancer. We demonstrate that following arrest of thymic export (either due to aging or adult thymectomy), T cells undergo a two-phase change in the expression of CD3 and CD5 molecules. Initial increase in CD5 and decrease in CD3 levels are consistent with preferential peripheral survival of T cells with relatively high avidity for self. These cells are prone for production of IL-10. Subsequently, the T cells reduce the levels of CD5 and become generally unresponsive (including the IL-10 production). Through both phases mice display reduced severity of an autoimmune disease EAE. These findings suggest that age-associated dysfunctions of the immune system could in part be due to functional erosion of T cells devised to protect the hosts from autoreactivity induced by T cells with high-avidity for self, that preferentially survive in mice with reduced thymic function (Figure 8).

## Methods

### Mice and in vivo manipulations

C57BL/6 mice were purchased from Taconic Farms (Germantown, NY). The generation of MTB TCRβ transgenic mice has been previously described [72]. All experiments using laboratory animals have been approved by the Institutional Animal Care and Use Committee.

For induction of EAE five mice per group were injected with an emulsion of MOG_38-50 _peptide ([73] solution in Complete Freund's adjuvant containing *Mycobacterium tuberculosis*, as described [74]. Mice also received 500 ng Pertussis toxin on days 0 and 2 relative to the encephalitogenic challenge. Mice were followed daily for clinical signs of the disease and were graded on the following basis: 0) no clinical signs; 1) flaccid tail; 2) hind limb paresis or partial paralysis; 3) total hind limb paralysis; 4) hind and front limb paralysis; and 5) moribund state or death.

### Quantitative PCR

Total RNA was isolated from cells using TRIzol followed by RNAse clean-up and treatment with DNAse I. Total RNA was reverse transcribed using the Superscript II RT kit and random hexamers as primers (Invitrogen, Carlsbad, CA). All PCR reactions were done in triplicates using ABI Prism 7700 Sequence Detector (Applied Biosystems, Piscataway, NJ), as previously described [23]. Briefly, TCRβ, IL-10, and 18SrRNA were amplified using TaqMan Universal PCR master mix (Applied Biosystems). The average threshold cycles (Ct) of the triplicates was used to compare the relative abundance of the mRNA. Ct of 18SrRNA was used to normalize all samples.

### Flow cytometry

Direct immunofluorescence staining was performed using following reagents: FITC conjugated anti-Vβ2, FITC- or APC-conjugated anti-CD5, FITC- or APC-conjugated anti-CD4, PerCP-conjugated anti-CD3, PE-conjugated anti-mouse CD8α, PE-conjugated anti-CD25 (all supplied by Pharmingen, San Diego, CA).

### Cell purification

Spleen cells were purified using pan T cell purification kit (Miltenyi Biotec, Bergisch Gladbach, Germany). Microbead labeled cells were negatively selected on magnetic cell separation (MACS) columns (Miltenyi Biotec), as per manufacturer instructions. Cell purity was generally 92-97%, as determined by flow cytometry. Intracellular staining for FoxP3 was performed using the anti-mouse/rat FoxP3 staining kit from eBioscience (San Diego, CA).

### In vitro stimulation assays

Spleen cells (2 × 10^5^/well) were incubated for 72 hours in flat bottom 96-well plates in the presence of various concentrations of purified anti-CD3 monoclonal antibody. Irradiated (2500 rads) WT spleen cells served as antigen presenting cells in cultures with thymocytes (5 × 10^5^/well), where indicated. During the last 8-16 hours of culture cells were pulsed with 0.5 μCi of ^3^H-thymidine (ICN Biomedicals, Costa Mesa, CA, USA) and thymidine incorporation was subsequently measured using a beta scintillation counter 1450 MicroBetaTM (Wallac, Turku, Finland).

### AICD analysis

T lymphocytes from WT or MTB TCR transgenic mice were isolated from the spleen by negative selection (Pan T Cell Isolation Kit, Miltenyi Biotec). T cells (10^6^/ml) were stimulated in vitro using CD3/CD28 beads (Dynabeads mouse CD3/CD28 T cell expander, Invitrogen) according to the manufacture's instruction. After 3 days, T cells were harvested and dead cells were removed by using gradient centrifugation. Viable T cells were then incubated for an additional 24 h with 10 U/ml recombinant mouse IL-2. For the secondary culture, T cells (5 × 10^5^/ml) were restimulated with anti mouse CD3 beads. Cells were then harvested and stained with Annexin-FITC and Propidium Iodide-PE using Annexin V-FITC apoptosis detection kit II (BD Biosciences) and CD4-allophycocyanin (BD Biosciences) according to the manufacturer's instructions. Analysis was based on a CD4 cell gate. Data were collected on a FACSCalibur (BD Biosciences) and analyzed using CellQuest software.

### Statistical analysis

Statistical significance of differences in the mean fluorescence intensities of CD5 and CD3 staining was calculated using Wilcoxon matched pairs test, performed using Graphpad Prism software, version 5.0a.

## Abbreviations

EAE: Experimental allergic encephalomyelitis

## Authors' contributions

RD and GLB performed and analyzed the activation induced cell death experiments. MS performed and analyzed real time PCR and T cell proliferation assays. DJ performed and analyzed IL-10 production and detection assays, and participated in drafting the manuscript. ZTC performed and analyzed all other experiments, and participated in drafting the manuscript. SV conceived the study and the manuscript and took part in writing the manuscript. All authors read and approved the final manuscript.

## References

[B1] Sadighi Akha AA Miller RA Signal transduction in the aging immune system Curr Opin Immunol 2005 17 486 491 10.1016/j.coi.2005.07.004 16061371

[B2] Miller RA The aging immune system: primer and prospectus Science 1996 273 70 74 10.1126/science.273.5271.70 8658199

[B3] Lerner A Yamada T Miller RA Pgp-1hi T lymphocytes accumulate with age in mice and respond poorly to concanavalin A Eur J Immunol 1989 19 977 982 10.1002/eji.1830190604 2666144

[B4] Miller RA Garcia G Kirk CJ Witkowski JM Early activation defects in T lymphocytes from aged mice Immunol Rev 1997 160 79 90 10.1111/j.1600-065X.1997.tb01029.x 9476667

[B5] Hirokawa K Age-related changes of signal transduction in T cells Exp Gerontol 1999 34 7 18 10.1016/S0531-5565(98)00067-9 10197724

[B6] Grossmann A Maggio-Price L Jinneman JC Rabinovitch PS Influence of aging on intracellular free calcium and proliferation of mouse T-cell subsets from various lymphoid organs Cell Immunol 1991 135 118 131 10.1016/0008-8749(91)90259-E 1826862

[B7] Thoman ML Weigle WO Lymphokines and aging: interleukin-2 production and activity in aged animals J Immunol 1981 127 2102 2106 6457862

[B8] Linton PJ Haynes L Klinman NR Swain SL Antigen-independent changes in naive CD4 T cells with aging J Exp Med 1996 184 1891 1900 10.1084/jem.184.5.1891 8920876 PMC2192899

[B9] Scollay RG Butcher EC Weissman IL Thymus cell migration. Quantitative aspects of cellular traffic from the thymus to the periphery in mice Eur J Immunol 1980 10 210 218 10.1002/eji.1830100310 7379836

[B10] Metcalf D Moulds R Pike B Influence of the spleen and thymus on immune responses in ageing mice Clin Exp Immunol 1967 2 109 120 4291612 PMC1578823

[B11] Hartwig M Steinmann G On a causal mechanism of chronic thymic involution in man Mech Ageing Dev 1994 75 151 156 10.1016/0047-6374(94)90083-3 7823637

[B12] Kadish JL Basch RS Hematopoietic thymocyte precursors. I. Assay and kinetics of the appearance of progeny J Exp Med 1976 143 1082 1099 10.1084/jem.143.5.1082 4575 PMC2190196

[B13] Freitas AA Rocha B Population biology of lymphocytes: the flight for survival Annu Rev Immunol 2000 18 83 111 10.1146/annurev.immunol.18.1.83 10837053

[B14] Mackall CL Gress RE Pathways of T-cell regeneration in mice and humans: implications for bone marrow transplantation and immunotherapy Immunol Rev 1997 157 61 72 10.1111/j.1600-065X.1997.tb00974.x 9255622

[B15] Linton PJ Dorshkind K Age-related changes in lymphocyte development and function Nat Immunol 2004 5 133 139 14749784 10.1038/ni1033

[B16] Ge Q Rao VP Cho BK Eisen HN Chen J Dependence of lymphopenia-induced T cell proliferation on the abundance of peptide/MHC epitopes and strength of their interaction with T cell receptors Proc Natl Acad Sci USA 2001 98 1728 1733 10.1073/pnas.98.4.1728 11172019 PMC29325

[B17] Moses CT Thorstenson KM Jameson SC Khoruts A Competition for self ligands restrains homeostatic proliferation of naive CD4 T cells Proc Natl Acad Sci USA 2003 100 1185 1190 10.1073/pnas.0334572100 12525694 PMC298748

[B18] Kieper WC Burghardt JT Surh CD A role for TCR affinity in regulating naive T cell homeostasis J Immunol 2004 172 40 44 14688307 10.4049/jimmunol.172.1.40

[B19] Azzam HS DeJarnette JB Huang K Emmons R Park CS Sommers CL El-Khoury D Shores EW Love PE Fine tuning of TCR signaling by CD5 J Immunol 2001 166 5464 5472 11313384 10.4049/jimmunol.166.9.5464

[B20] Smith K Seddon B Purbhoo MA Zamoyska R Fisher AG Merkenschlager M Sensory adaptation in naive peripheral CD4 T cells J Exp Med 2001 194 1253 1261 10.1084/jem.194.9.1253 11696591 PMC2195983

[B21] Kassiotis G Zamoyska R Stockinger B Involvement of avidity for major histocompatibility complex in homeostasis of naive and memory T cells J Exp Med 2003 197 1007 1016 10.1084/jem.20021812 12707300 PMC2193871

[B22] Perez-Villar JJ Whitney GS Bowen MA Hewgill DH Aruffo AA Kanner SB CD5 negatively regulates the T-cell antigen receptor signal transduction pathway: involvement of SH2-containing phosphotyrosine phosphatase SHP-1 Mol Cell Biol 1999 19 2903 2912 10082557 10.1128/mcb.19.4.2903 PMC84084

[B23] Stojakovic M Salazar-Fontana LI Tatari-Calderone Z Badovinac VP Santori FR Kovalovsky D Sant'Angelo D Harty JT Vukmanovic S Adaptable TCR avidity thresholds for negative selection J Immunol 2008 181 6770 6778 18981094 10.4049/jimmunol.181.10.6770

[B24] Turner SJ Doherty PC McCluskey J Rossjohn J Structural determinants of T-cell receptor bias in immunity Nat Rev Immunol 2006 6 883 894 10.1038/nri1977 17110956

[B25] Zeh HJI Perry-Lalley D Dudley ME Rosenberg SA Yang JC High avidity CTLs for two self antigens demonstrate superior in vitro and in vivo antitumor efficacy J Immunol 1999 162 989 994 9916724

[B26] Derby MA Alexander-Miller MA Tse R Berzofsky JA High-avidity CTL exploit two complementary mechanisms to provide better protection against viral infection than low-avidity CTL J Immunol 2001 166 1690 1697 11160212 10.4049/jimmunol.166.3.1690

[B27] Holler PD Chlewiski LK Kranz DM TCRs with high affinity for foreign pMHC show self reactivity Nature Immunol 2003 4 55 62 10.1038/ni863 12469116

[B28] Santori FR Brown SM Vukmanovic S Genomics-based identification of self-ligands with T cell receptor-specific biological activity Immunol Rev 2002 190 146 160 10.1034/j.1600-065X.2002.19011.x 12493012

[B29] Amrani A Verdaguer J Serra P Tafuro S Tan R Santamaria P Progression of autoimmune diabetes driven by avidity maturation of a T-cell population Nature 2000 406 739 742 10.1038/35021081 10963600

[B30] Garcia KC Radu CG Ho J Ober RJ Ward ES Kinetics and thermodynamics of T cell receptor- autoantigen interactions in murine experimental autoimmune encephalomyelitis Proc Nat Acad Sci USA 2001 98 6818 6823 10.1073/pnas.111161198 11391002 PMC34436

[B31] Gronski MA Boulter JM Moskophidis D Nguyen LT Holmberg K Elford AR Deenick EK Kim HO Penninger JM Odermatt B TCR affinity and negative regulation limit autoimmunity Nat Med 2004 10 1234 1239 10.1038/nm1114 15467726

[B32] Han B Serra P Yamanouchi J Amrani A Elliott J Dickie P Dilorenzo T Santamaria P Developmental control of CD8 T cell-avidity maturation in autoimmune diabetes J Clin Invest 2005 115 1879 1887 10.1172/JCI24219 15937548 PMC1142112

[B33] Li L Boussiotis VA Physiologic regulation of central and peripheral T cell tolerance: lessons for therapeutic applications J Mol Med 2006 84 887 899 10.1007/s00109-006-0098-5 16972086

[B34] Buer J Lanoue A Franzke A Garcia C von Boehmer H Sarukhan A Interleukin 10 secretion and impaired effector function of major histocompatibility complex class II restricted T cells anergized in vivo J Exp Med 1988 187 177 183 10.1084/jem.187.2.177 PMC2212096 9432975

[B35] Sundstedt A Hoiden I Rosendahl A Kalland T van Rooijen N Dohlsten M Immunoregulatory role of IL-10 during superantigen-induced hyporesponsiveness in vivo J Immunol 1997 158 180 186 8977189

[B36] Burkhart C Liu GY Anderton SM Metzler B Wraith DC Peptide-induced T cell regulation of experimental autoimmune encephalomyelitis: a role for IL-10 Int Immunol 1999 11 1625 1634 10.1093/intimm/11.10.1625 10508180

[B37] Seewaldt S Alferink J Forster I Interleukin-10 is crucial for maintenance but not for developmental induction of peripheral T cell tolerance Eur J Immunol 2002 32 3607 3616 10.1002/1521-4141(200212)32:12<3607::AID-IMMU3607>3.0.CO;2-O 12516547

[B38] Starr TK Jameson SC Hogquist KA Positive and negative selection of T cells Annu Rev Immunol 2003 21 139 176 10.1146/annurev.immunol.21.120601.141107 12414722

[B39] Palmer E The T-cell antigen receptor: a logical response to an unknown ligand J Recept Signal Transduct Res 2006 26 367 378 10.1080/10799890600919094 17118787

[B40] Vukmanovic S Neubert TA Santori FR Could TCR antagonism explain associations between MHC genes and disease? Trends Mol Med 2003 9 139 146 10.1016/S1471-4914(03)00029-7 12727139

[B41] Jameson SC T cell homeostasis: keeping useful T cells alive and live T cells useful Semin Immunol 2005 17 231 237 10.1016/j.smim.2005.02.003 15826828

[B42] Jabbari A Harty JT Cutting edge: differential self-peptide/MHC requirement for maintaining CD8 T cell function versus homeostatic proliferation J Immunol 2005 175 4829 4833 16210583 10.4049/jimmunol.175.8.4829

[B43] Oehen S Brduscha-Riem K Naive cytotoxic T lymphocytes spontaneously acquire effector function in lymphocytopenic recipients: a pitfall for T cell memory studies? EurJImmunol 1999 29 608 614 10.1002/(SICI)1521-4141(199902)29:02<608::AID-IMMU608>3.0.CO;2-A 10064077

[B44] Kieper WC Jameson SC Homeostatic expansion and phenotypic conversion of naive T cells in response to self peptide/MHC ligands Proc Natl Acad Sci USA 1999 96 13306 13311 10.1073/pnas.96.23.13306 10557316 PMC23943

[B45] Goldrath AW Bogatzki LY Bevan MJ Naive T cells transiently acquire a memory-like phenotype during homeostasis-driven proliferation JExpMed 2000 192 557 564 10.1084/jem.192.4.557 PMC2193243 10952725

[B46] Cho BK Rao VP Ge Q Eisen HN Chen J Homeostasis-stimulated proliferation drives naive T cells to differentiate directly into memory T cells JExpMed 2000 192 549 556 10.1084/jem.192.4.549 PMC2193235 10952724

[B47] Murali-Krishna K Ahmed R Cutting edge: naive T cells masquerading as memory cells J Immunol 2000 165 1733 1737 10925249 10.4049/jimmunol.165.4.1733

[B48] Baccala R Theofilopoulos AN The new paradigm of T-cell homeostatic proliferation-induced autoimmunity Trends Immunol 2005 26 5 8 10.1016/j.it.2004.11.006 15629402

[B49] Hochberg MC Wallace DJ, Hahn BH The epidemiology of systemic lupus erythematosus Dubois' Lupus Erythematosus 1997 Baltimore: Williams & Wilkins 4922357259

[B50] Weinshenker BG Bass B Rice GP Noseworthy J Carriere W Baskerville J Ebers GC The natural history of multiple sclerosis: a geographically based study. 2. Predictive value of the early clinical course Brain 1989 112 1419 1428 10.1093/brain/112.6.1419 2597989

[B51] Castle SC Clinical relevance of age-related immune dysfunction Clin Inf Dis 2000 31 578 585 10.1086/313947 10987724

[B52] Stojakovic M Tatari-Calderone Z Maric C Hoang A Vukmanovic S Paradoxical arrest in lupus activity in BXSB mice with highly autoreactive T cells Lupus 2010 19 182 191 10.1177/0961203309350756 19946033

[B53] Zepp J Wu L Li X IL-17 receptor signaling and T helper 17-mediated autoimmune demyelinating disease Trends Immunol 2011 32 232 239 10.1016/j.it.2011.02.007 21493143 PMC3329781

[B54] Astier AL Meiffren G Freeman S Hafler DA Alterations in CD46-mediated Tr1 regulatory T cells in patients with multiple sclerosis J Clin Invest 2006 116 3252 3257 10.1172/JCI29251 17099776 PMC1635165

[B55] Martinez-Forero I Garcia-Munoz R Martinez-Pasamar S Inoges S Lopez-Diaz de Cerio A Palacios R Sepulcre J Moreno B Gonzalez Z Fernandez-Diez B IL-10 suppressor activity and ex vivo Tr1 cell function are impaired in multiple sclerosis Eur J Immunol 2008 38 576 586 10.1002/eji.200737271 18200504

[B56] Hobb sMV Weigle WO Ernst DN Interleukin-10 production by splenic CD4+ cells and cell subsets from young and old mice Cell Immunol 1994 154 264 272 10.1006/cimm.1994.1076 7510582

[B57] Kuhn R Lohler J Rennick D Rajewsky K Muller W Interleukin-10-deficient mice develop chronic enterocolitis Cell 1993 75 263 274 10.1016/0092-8674(93)80068-P 8402911

[B58] Groux H Cottrez F The complex role of interleukin-10 in autoimmunity J Autoimmun 2003 20 281 285 10.1016/S0896-8411(03)00044-1 12791313

[B59] Rott O Fleischer B Cash E Interleukin-10 prevents experimental allergic encephalomyelitis in rats Eur J Immunol 1994 24 1434 1440 10.1002/eji.1830240629 7515815

[B60] Nagelkerken L Blauw B Tielemans M IL-4 abrogates the inhibitory effect of IL-10 on the development of experimental allergic encephalomyelitis in SJL mice Int Immunol 1997 9 1243 1251 10.1093/intimm/9.9.1243 9310827

[B61] Cannella B Gao YL Brosnan C Raine CS IL-10 fails to abrogate experimental autoimmune encephalomyelitis J Neurosci Res 1996 45 735 746 10.1002/(SICI)1097-4547(19960915)45:6<735::AID-JNR10>3.0.CO;2-V 8892085

[B62] Bettelli E Das MP Howard ED Weiner HL Sobel RA Kuchroo VK IL-10 is critical in the regulation of autoimmune encephalomyelitis as demonstrated by studies of IL-10- and IL-4-deficient and transgenic mice J Immunol 1998 161 3299 3306 9759845

[B63] Segal BM Dwyer BK Shevach EM An interleukin (IL)-10/IL-12 immunoregulatory circuit controls susceptibility to autoimmune disease J Exp Med 1998 187 537 546 10.1084/jem.187.4.537 9463404 PMC2212155

[B64] Samoilova EB Horton JL Chen Y Acceleration of experimental autoimmune encephalomyelitis in interleukin-10-deficient mice: roles of interleukin-10 in disease progression and recovery Cell Immunol 1998 188 118 124 10.1006/cimm.1998.1365 9756642

[B65] Beebe AM Cua DJ de Waal Malefyt R The role of interleukin-10 in autoimmune disease: systemic lupus erythematosus (SLE) and multiple sclerosis (MS) Cytokine Growth Factor Rev 2002 13 403 412 10.1016/S1359-6101(02)00025-4 12220553

[B66] Llorente L Richaud-Patin Y García-Padilla C Claret E Jakez-Ocampo J Cardiel MH Alcocer-Varela J Grangeot-Keros L Alarcón-Segovia D Wijdenes J Clinical and biologic effects of anti-interleukin-10 monoclonal antibody administration in systemic lupus erythematosus Arthritis Rheum 2000 43 1790 1800 10.1002/1529-0131(200008)43:8<1790::AID-ANR15>3.0.CO;2-2 10943869

[B67] Ishida H Muchamuel T Sakaguchi S Andrade S Menon S Howard M Continuous administration of anti-interleukin 10 antibodies delays onset of autoimmunity in NZB/W F1 mice J Exp Med 1994 179 305 310 10.1084/jem.179.1.305 8270873 PMC2191319

[B68] Yin Z Bahtiyar G Zhang N Liu L Zhu P Robert ME McNiff J Madaio MP Craft J IL-10 regulates murine lupus J Immunol 2002 169 2148 2155 12165544 10.4049/jimmunol.169.4.2148

[B69] Blenman KR Duan B Xu Z Wan S Atkinson MA Flotte TR Croker BP Morel L IL-10 regulation of lupus in the NZM2410 murine model Lab Investig 2006 86 1136 1148 16924244 10.1038/labinvest.3700468

[B70] Ely KH Roberts AD Kohlmeier JE Blackman MA Woodland DL Aging and CD8+ T cell immunity to respiratory virus infections Exp Gerontol 2007 42 427 431 10.1016/j.exger.2006.11.017 17197143 PMC1964788

[B71] Janicki CN Jenkinson SR Williams NA Morgan DJ Loss of CTL function among high-avidity tumor-specific CD8+ T cells following tumor infiltration Cancer Res 2008 68 2993 3000 10.1158/0008-5472.CAN-07-5008 18413769

[B72] Santori FR Popmihajlov Z Badovinac VP Smith C Radoja S Harty JT Vukmanovic S TCRβ chain that forms peptide-independent alloreactive TCR transfers reduced reactivity with irrelevant peptide/MHC complex J Immunol 2007 178 6109 6114 17475836 10.4049/jimmunol.178.10.6109

[B73] Hilliard B Samoilova EB Liu TS Rostami A Chen Y Experimental autoimmune encephalomyelitis in NF-kappa B-deficient mice:roles of NF-kappa B in the activation and differentiation of autoreactive T cells J Immunol 1999 163 2937 2943 10453042

[B74] Oliver AR Lyon GM Ruddle NH Rat and human myelin oligodendrocyte glycoproteins induce experimental autoimmune encephalomyelitis by different mechanisms in C57BL/6 mice J Immunol 2003 171 462 468 12817031 10.4049/jimmunol.171.1.462

